# The Application of e-Mental Health in Response to COVID-19: Scoping Review and Bibliometric Analysis

**DOI:** 10.2196/32948

**Published:** 2021-12-06

**Authors:** Louise A Ellis, Isabelle Meulenbroeks, Kate Churruca, Chiara Pomare, Sarah Hatem, Reema Harrison, Yvonne Zurynski, Jeffrey Braithwaite

**Affiliations:** 1 Centre for Healthcare Resilience and Implementation Science Australian Institute of Health Innovation Macquarie University Sydney Australia; 2 National Health and Medical Research Council Partnership Centre for Health System Sustainability Australian Institute of Health Innovation Macquarie University Sydney Australia

**Keywords:** e-mental health, mental health, COVID-19, bibliometrics, health systems

## Abstract

**Background:**

The COVID-19 pandemic and its mitigation measures and impacts, such as shelter-in-place orders, social isolation, restrictions on freedoms, unemployment, financial insecurity, and disrupted routines, have led to declines in mental health worldwide and concomitant escalating demands for mental health services. Under the circumstances, electronic mental health (e-mental health) programs and services have rapidly become the “new normal.”

**Objective:**

The aim of this study was to assess key characteristics and evidence gaps in the e-mental health literature published in relation to the COVID-19 pandemic via a scoping review and bibliometric analysis.

**Methods:**

We conducted a search of four academic databases (ie, MEDLINE, Embase, PsycInfo, and CINAHL) for documents published from December 31, 2019, to March 31, 2021, using keywords for e-mental health and COVID-19. Article information was extracted that was relevant to the review objective, including journal, type of article, keywords, focus, and corresponding author. Information was synthesized by coding these attributes and was then summarized through descriptive statistics and narrative techniques. Article influence was examined from Altmetric and CiteScore data, and a network analysis was conducted on article keywords.

**Results:**

A total of 356 publications were included in the review. Articles on e-mental health quickly thrived early in the pandemic, with most articles being nonempirical, chiefly commentaries or opinions (n=225, 63.2%). Empirical publications emerged later and became more frequent as the pandemic progressed. The United States contributed the most articles (n=160, 44.9%), though a notable number came from middle-income countries (n=59, 16.6%). Articles were spread across 165 journals and had above-average influence (ie, almost half of the articles were in the top 25% of output scores by Altmetric, and the average CiteScore across articles was 4.22). The network analysis of author-supplied keywords identified key topic areas, including specific mental disorders, eHealth modalities, issues and challenges, and populations of interest. These were further explored via full-text analysis. Applications of e-mental health during the pandemic overcame, or were influenced by, system, service, technology, provider, and patient factors.

**Conclusions:**

COVID-19 has accelerated applications of e-mental health. Further research is needed to support the implementation of e-mental health across system and service infrastructures, alongside evidence of the relative effectiveness of e-mental health in comparison to traditional modes of care.

## Introduction

Mental illness is widespread worldwide. Depression and anxiety are the two most common mental health conditions, affecting 4.4% and 3.6% of the world’s adult population, respectively [1]. These disorders lead to considerable losses in health and functioning, with depressive disorders ranked as the single largest contributor to nonfatal health loss globally [1]. At its worst, depression can lead to suicide; more than 800,000 people die by suicide every year along with many more attempts [1,2].

COVID-19 was first reported in Wuhan, China, on December 31, 2019, and on March 11, 2020, the World Health Organization declared the disease a global pandemic [3,4]. By April 2020, most countries worldwide had introduced stay-at-home lockdown and quarantine measures to contain the disease [5]. The pandemic and the public health measures implemented to slow the spread of COVID-19 heightened risk factors associated with poor mental health, including financial insecurity, unemployment, and fear. During this time, access to protective mental health factors, such as social connection, employment, access to physical exercise, and access to health services, fell considerably [6,7].

Mental health data suggest that COVID-19 has exacerbated the mental health crisis [8], with the situation being described by some as a global mental health “catastrophe” [9]. The strictly implemented stay-at-home and quarantine measures are reported to have exacerbated stress and anger, substance abuse, online gaming, and gambling, and has led to a rise in rates of domestic violence and sexual abuse in the general population [7]. In Australia, one study identified that the population prevalence of poor mental health more than doubled from 20% [10] to around 45% in the first year of the pandemic [11], with similar increases being reported in other countries internationally [12,13]. Latest figures suggest that the groups most affected include young people, those living alone, those with lower socioeconomic status, and those who became unemployed as a result of the pandemic [8]. Although the uptake of COVID-19 vaccines is expected to reduce the likelihood of further restrictions and lockdowns, thereby alleviating some immediate stressors, much of the additional mental health burden is expected to persist due to the economic impacts and trauma resulting directly and indirectly from the pandemic.

**Figure 1:**
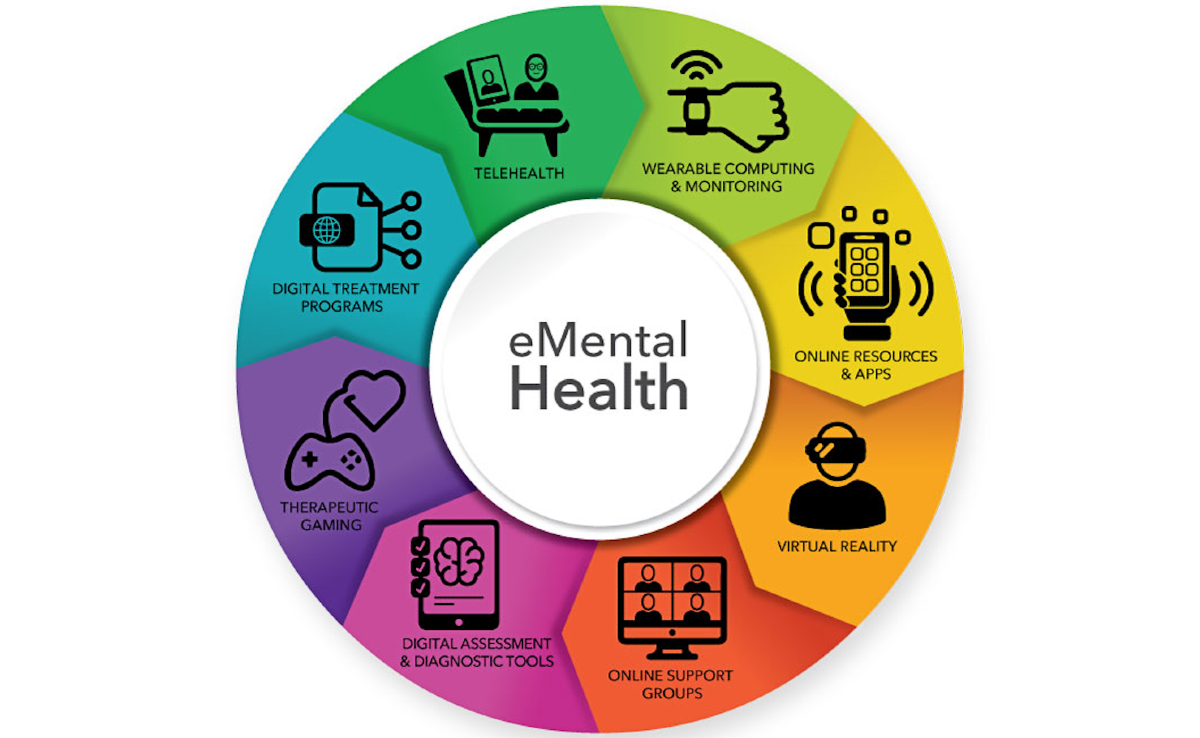
Electronic mental health (e-mental health) technologies [18].

As the COVID-19 pandemic continues, electronic mental health (e-mental health) is rapidly becoming the “new normal” [14]. The opportunities afforded by programs and services that aim to treat and manage mental health problems have been recognized for over two decades [15,16]. e-Mental health is broadly defined as “mental health services and information delivered or enhanced through the internet and related technologies. It includes all technology-enabled therapies, including internet-based programs, mobile phone applications, telehealth and informational websites” (page 475 of Stone and Waldron) [17]. As a result of rapid technological developments over the past decade, the growing field of e-mental health covers far more than telehealth, with the field now ranging from online support groups and resources to digital assessment and treatment programs and, more recently, to therapeutic gaming and virtual reality (Figure 1) [18]. Telehealth and the associated terms telemedicine and telecare [19-21] historically focused on service provision via telephone, yet the technological advancement that has enabled telehealth to be delivered via communication software, including videoconferencing, has blurred the boundaries between eHealth, mobile health (mHealth), and telehealth. For the purposes of this review, we, therefore, use the phrase e-mental health as an umbrella term to capture eHealth, mHealth, and telehealth [22].

With the onset of COVID-19, the potential benefits of the use of e-mental health programs have been greatly reinforced by the need for mental health services to adapt to social distancing and stay-at-home measures. This has propelled e-mental health into widespread use in developed countries in favor of face-to-face therapies. By mid-2020, more than 80% of high-income countries had shifted to e-mental health technologies to replace or supplement in-person mental health consultations [8]. For example, by the end of 2020, Kaiser Permanente, the largest managed care organization in the United States with 12 million members, was delivering 90% of its psychiatric care virtually [23,24]. For the most part, governments in charge of publicly funded care systems have been responsive in ensuring the availability of e-mental health to the population, in some cases by adding new entitlements to services [9]. For example, in the United States, on March 17, 2020, the Centers for Medicare & Medicaid Services relaxed several requirements for the provision of and payment for telehealth services to Medicare patients [25]. Various countries have also introduced new legal and practice guidelines to assure patient privacy and quality of care [26,27]. In India, the Medical Council released the *Telemedicine practice guidelines* in May 2020 to help remove some of the administrative and legal concerns present in practicing virtual care [27-29]. The COVID‑19 pandemic has also pushed some governments to make online or digital mental health resources widely available to the general population. As another example, the Government of Canada launched a new portal for mental health resources, Wellness Together Canada, which offers a no-cost wellness self-assessment, tracking and support resources, and counseling by text or telephone [30].

**Figure 2:**
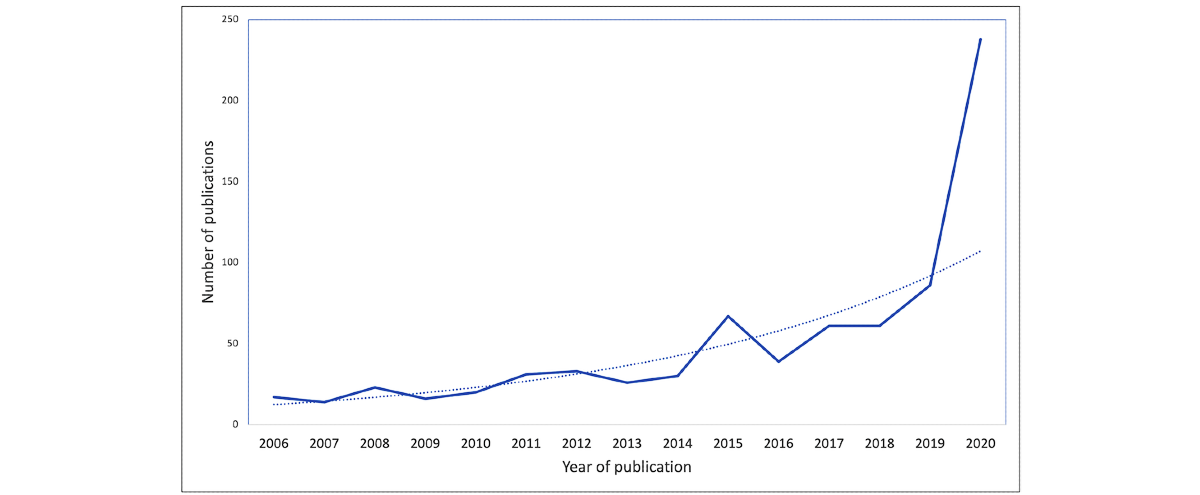
Increase in publications during 2020 (blue) and trendline (dotted blue), based on publications in MEDLINE using the search term “telepsychiatry” in titles or abstracts.

In this study, e-mental health evidence has accelerated since the onset of the COVID-19 pandemic, as indicated by the sharp increase in the number of publications in “telepsychiatry” in 2020 (Figure 2). With this burgeoning field, there is a need to identify key emerging issues in light of COVID-19 and to examine developing consensus, gaps in knowledge, as well as pertinent areas for future research. This study involved a combined scoping review and bibliometric analysis of the e-mental health literature since the beginning of the COVID-19 pandemic, exploring characteristics, key topics, and the influence of publications. Our specific objectives were as follows:

1. Investigate the key characteristics of publications in the e-mental health literature since the start of the COVID-19 pandemic (ie, from 2020 onward).
2. Identify the key topics covered by this body of literature in order to determine potential evidence gaps.
3. Analyze characteristics in the most influential publications, as indicated by Altmetric and CiteScore data.
4. Examine the future directions for research and practice in the e-mental health field, both during the COVID-19 pandemic and beyond.

## Methods

### Overview

The review followed a predetermined protocol, developed in accordance with the PRISMA-ScR (Preferred Reporting Items for Systematic Reviews and Meta-Analyses extension for Scoping Reviews) guidelines [31,32] and the Joanna Briggs Institute Methodology for Scoping Reviews [33]. A scoping review method was used to examine the extent, range, and nature of work on this topic; identify gaps; and provide suggestions to improve future directions for research and practice on e-mental health [34]. Quality assessments were not undertaken, as the aim was to examine the full breadth of the literature, consistent with the general aims and methodology of scoping reviews [35].

### Search Strategy

Four academic databases (ie, MEDLINE, Embase, PsycInfo, and CINAHL) were searched from December 31, 2019, to March 31, 2021. The search strategy consisted of terms pertaining to eHealth (eg, “eHealth” and “telehealth”), more specific terms related to e-mental health (eg, “m-mental health”), common mental disorders (eg, “anxiety” and “depression”), and COVID-19. The search strategy was adapted for each database as necessary (see Multimedia Appendix 1 for the complete search strategy, using Ovid MEDLINE as an example). The search strategy was developed in consultation with an academic research librarian and was reviewed by all authors prior to execution.

### Inclusion and Exclusion Criteria

Articles were included if they were (1) in the English language, (2) peer-reviewed journal articles, (3) discussed the application of e-mental health (theoretical or applied), and (4) were published following the onset of COVID-19 (from December 31, 2019). No restrictions were placed on the target population or setting. Studies were excluded if they did not focus on e-mental health, they were book chapters or conference proceedings, or the full text was not in English.

### Citation Screening

Reference details, including abstracts, were downloaded into the reference management software EndNote X9 (Clarivate), exported to Microsoft Excel, and divided among the research team for title and abstract screening. Two reviewers (IM and LAE) independently reviewed 5% of the titles and abstracts; interrater reliability between the two reviewers was high (κ=0.95, 95% CI 0.77-1.00; *P*<.001). Full-text screening was conducted by four independent reviewers (LAE, IM, KC, and Tamasha Jayawardena). A total of 5% of full texts were reviewed by four reviewers, with the interrater reliability assessed to be sufficiently high (κ≥0.80) [36].

### Data Extraction

A customized data extraction workbook was developed in Microsoft Excel. The workbook was piloted by each of the four reviewers with a subset of papers (n=10). Issues in consistency of data entry and usability of the template were then discussed, and modifications were made accordingly. Key information extracted included the following: article characteristics (ie, authors, date of publication, country of residence of the corresponding author, and journal name); article keywords, as supplied by the authors of the paper; and article type (ie, empirical, nonempirical, protocol, or review). For empirical studies (ie, studies that present an analysis of primary or secondary data in their results), information on study design (ie, quantitative, qualitative, or mixed methods) and methods (eg, surveys and interviews) were also extracted. In addition, article titles, abstracts, and keywords were searched for terms to identify papers with specific subpopulations of interest (eg, children, adolescents, veterans, and the elderly) and specific mental disorders (eg, depression, anxiety, posttraumatic stress disorder [PTSD], and substance abuse), as well as articles with a focus on issues or challenges related to the use or uptake of e-mental health in the context of COVID-19 (eg, barriers, privacy, and ethics).

Altmetric data were selected as the most appropriate measure of an article’s impact or influence because of the recency of this literature [37]; while citations have a lag effect, the real-time update of social media metrics makes Altmetric events around research outputs visible within a short amount of time (eg, hours or days). The Altmetric Attention Score, a composite score of social interest that includes mentions in newsfeeds, Twitter, Facebook, and Google, among other sources, was collected for each included study, where available, from Altmetric Explorer in June 2021. In addition, we recorded the CiteScore for the journal in which each article was published, taken from the Scopus database in June 2021. CiteScore metrics were used rather than the Journal Impact Factor, as CiteScore values give “a more comprehensive, transparent, and current view of a journal’s impact” (page 941 of Roldan-Valadez et al) [38], and they are provided for many more journals than the Journal Impact Factor. Although previous research indicates that an article’s Altmetric score within its first year of publication reliably predicts its future citation count, we followed the recommended optimal approach of using both Altmetric scores and CiteScore values as an indicator of article impact [39].

### Data Synthesis and Analysis

Articles were grouped together based on common attributes in the data (eg, article type). The country of the corresponding author was coded by income classification based on the World Bank’s definitions of gross national income per capita per year. The three categories were low (<US $1045), middle (US $1046-$12,695), and high income (>US $12,696) [40].

Key topic areas were identified through an analysis of article keywords. These were extracted by the research team and were then cleaned and checked for consistency. Derivative terms (eg, “health care” and “healthcare”) were amalgamated. Each keyword was reviewed and inductively classified by two authors (LAE and IM) into key topic and subtopic areas using the structure and definitions outlined in Multimedia Appendix 2. The keyword data were analyzed for frequency and co-occurrence and were graphically presented using Gephi (version 0.9.2) [41]. Our presentation of keyword data and discussion of key topic areas focused on the most influential papers as representatives of salient topic areas. To select these papers, we used a combination of Altmetric and CiteScore data, including publications with journal CiteScore values of ≥5, representing the top one-third of journals in our included articles, and/or Altmetric Attention Score values in the top 25% of all publications scored by Altmetric.

## Results

### Overview

**Figure 3:**
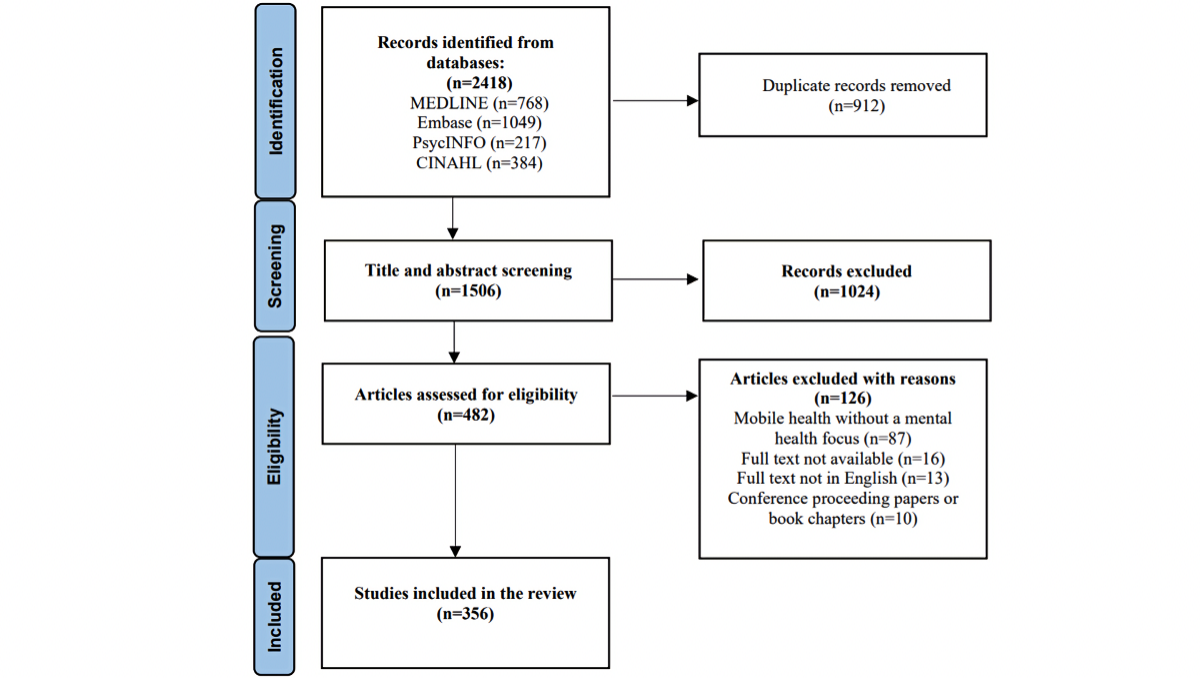
Search and review strategy.

The search retrieved a total of 2418 publications. After removing duplicates, 1506 remained for title and/or abstract review. Following title and/or abstract screening, 1023 publications were discarded as they did not meet the inclusion criteria. Based on the full-text assessment, a further 127 publications did not meet the inclusion criteria, resulting in 356 publications included in this review (see Multimedia Appendix 3 for included articles). Figure 3 demonstrates the inclusion and exclusion of papers at each stage of the screening process.

### Summary Characteristics and Bibliometric Properties of the Included Publications

**Figure 4:**
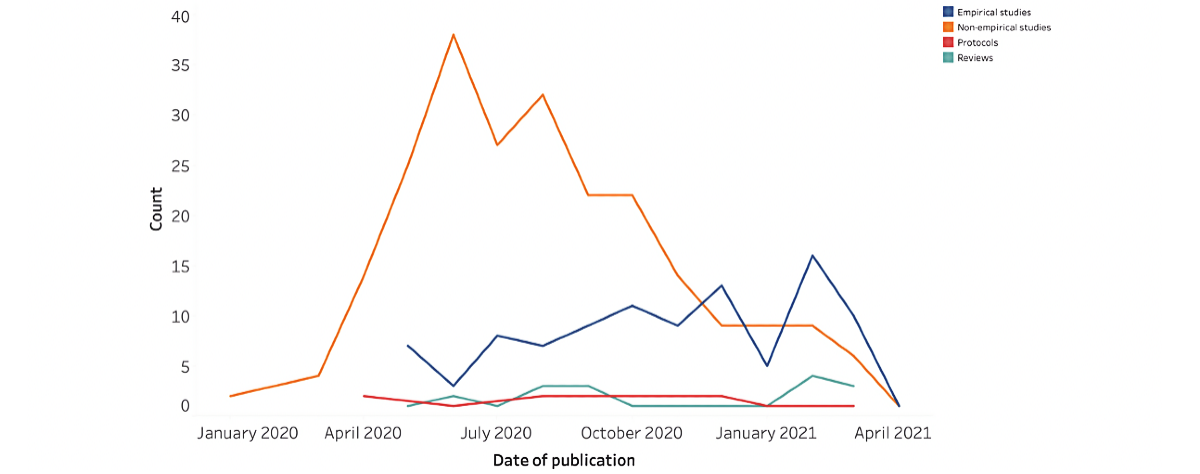
Date of publication by article type.

A summary of the key characteristics of the included articles is presented in Table 1. Of the 356 articles, most were nonempirical commentaries or opinions (n=225, 63.2%), around one-quarter were empirical publications (n=99, 27.8%), 20 were literature reviews (5.6%), and 12 were protocols (3.4%). Figure 4 shows the trends in article types published during the pandemic, with nonempirical publications peaking in June 2020 and empirical publications emerging later and becoming more frequent as the pandemic progressed.

**Table 1:** Summary of key characteristics of included publications.

Classification		Papers (N=356), n (%)^a^
**Country of corresponding author**		
	United States	160 (44.9)
	Australia	28 (7.9)
	India	27 (7.6)
	United Kingdom	20 (5.6)
	Canada	17 (4.8)
	Other	104 (9.2)
**Country income classification of corresponding author**		
	High income (>US $12,696)	297 (83.4)
	Middle income (US $1046-$12,695)	59 (16.6)
	Low income (<US $1045)	0 (0)
**Publication type and study methods**		
	Nonempirical	225 (63.2)
	Empirical	99 (27.8)
	Review	20 (5.6)
	Protocol	12 (3.4)
**Empirical study methods**		
	Quantitative methods	68 (68.7)
	Mixed methods	21 (21.2)
	Qualitative methods	10 (10.1)
**Mental disorder of focus**		
	Anxiety (including posttraumatic stress disorder)	71 (19.9)
	Depression	48 (13.5)
	Substance abuse disorders (including addiction)	17 (4.8)
	Psychotic disorders (including schizophrenia)	16 (4.5)
	Suicide	13 (3.7)
	Eating disorders	11 (3.1)
**Specific population of focus**		
	Children and/or adolescents	90 (25.3)
	Older adults and/or the elderly	20 (5.6)
	Veterans	17 (4.8)
	Health professionals	15 (4.2)
**Modality of focus**		
	Telephone or videoconferencing	142 (39.9)
	Smartphone apps	43 (12.1)
	Support groups	6 (1.2)

^a^Columns may not equal 356 due to missing values and overlap in some categories.

### Publication Types and Study Methods

Of the 225 nonempirical publications, most were commentaries or opinion pieces (n=106, 47.1%), with a further 49 (21.8%) classified as descriptive case studies, 36 (16.0%) as letters to the editor, 22 (9.8%) as unstructured reviews, 10 (4.4%) as editorials, and 2 (0.9%) as unstructured protocols (Table 1). Of the 99 empirical publications, the majority used quantitative methods (n=68, 69%), 21 (21%) used a mixed methods design, and 10 (10%) employed qualitative methods alone. Most of the quantitative and mixed methods studies (n=89, 39.6%) were cross-sectional (n=60, 67%), with only 4 (4%) randomized controlled trials [42-45] and 3 (3%) nonrandomized controlled trials identified [46-48].

Overall, the empirical studies focused on the uptake and effectiveness of e-mental health initiatives in providing mental health care during COVID-19. The nonempirical descriptive case studies outlined the processes and challenges of the rapid conversion to telehealth for a particular mental health service, while nonempirical commentary or opinion pieces more broadly outlined policy changes, issues, and challenges to e-mental health and offered guidance to clinicians (see Table 2 [49-52] for exemplar papers for each category).

**Figure 5:**
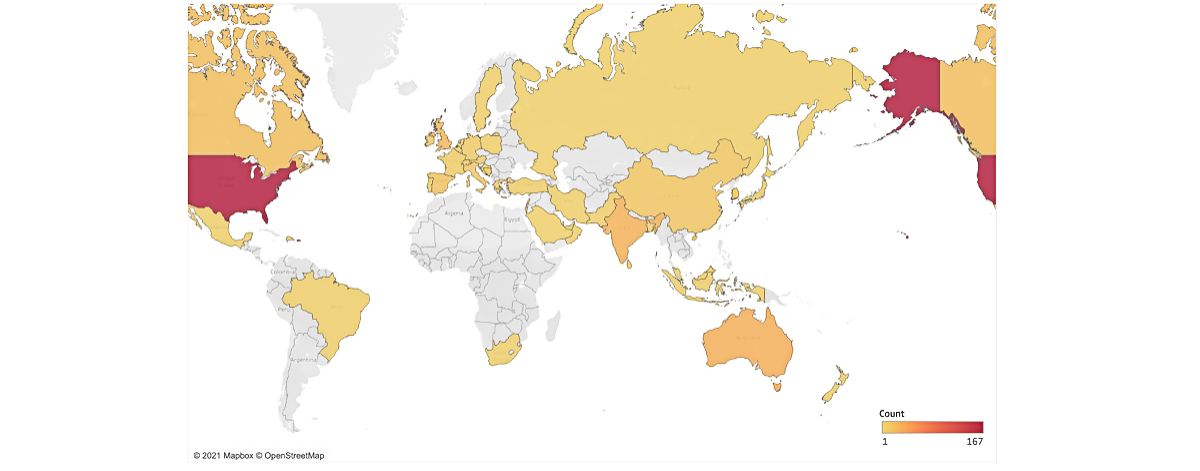
Global trends for publishing articles on electronic mental health applied to COVID-19.

Corresponding authors were predominantly from high-income countries, with almost half of the 356 included outputs coming from the United States (n=160, 44.9%), followed by Australia (n=28, 7.9%), the United Kingdom (n=20, 5.6%), and Canada (n=17, 4.8%). Notably, our study identified a number of publications from middle-income countries (n=59, 16.6%), with most of these being from India (n=29, 7.6%) and China (n=9, 2.5%) (Figure 5).

**Table 2:** Examples of included studies.

Authors (year)	Country	Article type	Article classification	Specific populations	Specific disorders	Article aim
Pierce et al (2020) [49]	United States	Empirical	Quantitative survey of 2691 psychologists	Veterans	Anxiety	To examine the amount of psychologists’ telepsychology use before the COVID-19 pandemic, during the pandemic, and anticipated use after the pandemic, as well as the demographic, training, policy, and clinical practice predictors of these changes
Steinberg et al (2020) [50]	United States	Empirical	Quantitative and qualitative survey of 51 pediatric psychologists	Children and adolescents	General	To examine the uptake of transitioning pediatric psychology services to meet mental health needs in response to a worldwide public health crisis, and to call attention to psychologists’ perceived benefits and challenges related to providing pediatric mental health services during the pandemic
Patel et al (2020) [51]	Ireland	Nonempirical	Descriptive case study	The elderly	General	To discuss the transition and challenges faced in rapidly implementing telehealth in a rural psychiatry-of-old-age service in the northwest of Ireland
Haque (2020) [52]	United States	Nonempirical	Perspective	Mental health professionals	General	To discuss policy changes due to the COVID-19 pandemic and to highlight what mental health providers should consider for future delivery and implementation of telehealth programs

### Publication Data

The 356 included articles were spread across 165 different journals, which were primarily focused on mental health, but varied in scope. Most of the journals had a reported CiteScore in Scopus (n=138, 83.6%), with scores ranging from 0.3 (Psychiatric Times) to 25.2 (The Lancet Psychiatry). A total of 3 articles (1.8%) were published in The Lancet Psychiatry [53-55], of which 2 were from middle-income countries [53,54]. The largest number of included articles were published in JMIR Mental Health (n=19, 11.5%; CiteScore=1.3), followed by the Asian Journal of Psychiatry (n=14, 8.4%; CiteScore=4.7) and the Indian Journal of Psychological Medicine (n=13, 7.9%; CiteScore=1.8), with one-quarter of articles (n=40, 24.2%) being published across the JMIR suite of journals (mean CiteScore 3.37). The mean CiteScore value across articles (ie, CiteScore for each article based on its journal CiteScore and then averaged across articles) was 4.22 (SD 3.53); however, CiteScore values varied by article type, with protocol papers having a lower average CiteScore (mean 2.1, SD 1.55) than empirical studies (mean 3.8, SD 2.06), nonempirical articles (mean 4.6, SD 4.16), and reviews (mean 4.4, SD 1.91).

Of the 242 publications with reported Altmetric data, almost half were ranked in the top 25% of all outputs scored by Altmetric (n=118, 48.8%). Altmetric Attention Score values ranged from 1 to 282 (mean 13.33, SD 30.04); though similar to CiteScore values, there was variation in Altmetric metrics by article type, with empirical studies (mean 14.10, SD 29.22), nonempirical articles (mean 13.6, SD 32.77), and reviews (mean 11.9, SD 14.11) having higher Altmetric scores than protocol papers (mean 5.4, 4.39). The publication with the highest Altmetric score was a nonempirical contribution by Kozloff et al, which outlined the adverse mental health consequences and virtual mental health service delivery options for people with schizophrenia during COVID-19, with an Altmetric Attention Score of 282, placing it in the 98th percentile of outputs of the same age [56]. The top five articles with the highest Altmetric Attention Score values are shown in Table 3 [24,42,55-57].

**Table 3:** The most influential publications based on Altmetric data.

Authors (year)	Country	Title	Journal	Altmetric Attention Score^a^	CiteScore^b^	Article type
Kozloff et al (2020) [56]	Canada	The COVID-19 global pandemic: Implications for people with schizophrenia and related disorders	Schizophrenia Bulletin	282	12.2	Nonempirical
Torous et al (2020) [24]	United States	Digital mental health and COVID-19: Using technology today to accelerate the curve on access and quality tomorrow	JMIR Mental Health	197	1.3	Nonempirical
Ben-Zeev et al (2020) [42]	United States	Augmenting evidence-based care with a texting mobile interventionist: A pilot randomized controlled trial	Psychiatric Services	146	4.6	Empirical
Rahman et al (2020) [55]	United Kingdom	The NIMH^c^ global mental health research community and COVID-19	The Lancet Psychiatry	124	25.2	Nonempirical
Zhou et al (2020) [57]	Australia	The role of telehealth in reducing the mental health burden from COVID-19	Telemedicine Journal and e-Health	124	4.6	Nonempirical

^a^Altmetric data from Altmetric Explorer as of June 2021. All scores were in the top 5%.

^b^CiteScore in Scopus as of June 2021.

^c^NIMH: National Institute of Mental Health.

### High-Impact Publications and Keyword Analysis

The topmost influential publications—defined as those with Altmetric Attention Score values in the top 25% of all research scored by Altmetric, and/or CiteScore values of ≥5—were identified. Based on this definition, close to half of our 356 included publications were classified as influential (n=165, 46.3%). The 165 influential articles were broadly reflective of the countries and country incomes previously reported (high-income countries defined here: n=143, 86.7%).

**Figure 6:**
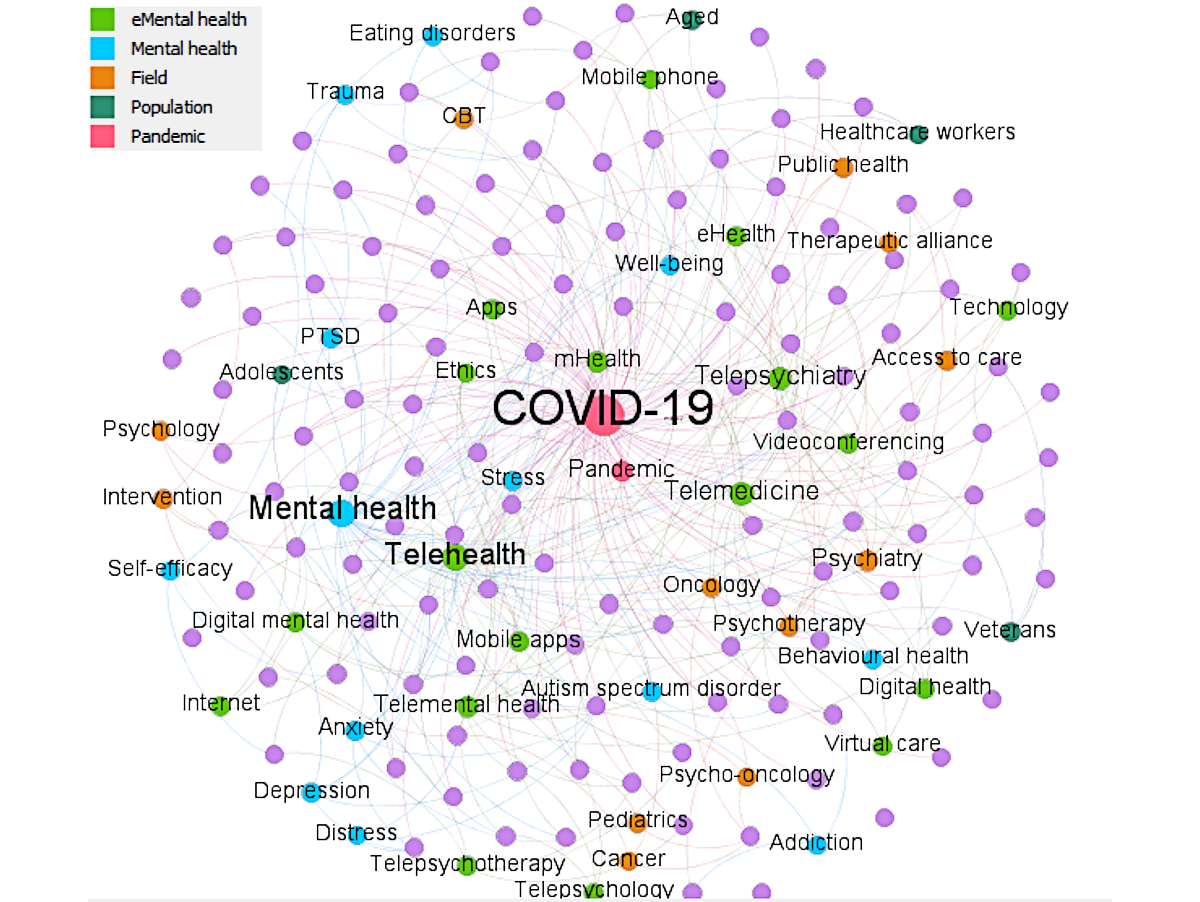
Network of co-occurring keywords in 165 of the publications with Altmetric values in the top 25% or CiteScore values of ≥5. Each circle (node) is a keyword, and each line (edge) represents co-occurrence. The size of each node indicates the number of times a keyword was used. Colors represent different topic areas. CBT: cognitive behavioral therapy; mHealth: mobile health; PTSD: posttraumatic stress disorder.

Among the 165 most influential publications, 124 had author-contributed keywords, with a total of 273 unique keywords in this subset of papers. The frequency with which these keywords were used together on a publication is visually depicted in the network of co-occurring keywords (Figure 6). In this network, each circle (node) represents a keyword, and each line (edge) indicates co-occurrence on a publication. The most common keywords (highest in-degree score), as reflected in size in the figure, were as follows: COVID-19 (n=90), mental health (n=37), telehealth (n=32), telemedicine (n=30), and telepsychiatry (n=17). The sociogram includes 124 papers (grey nodes), 50 keywords (colored nodes), and 390 edges. The size of each node is filtered by the in-degree score, where bigger nodes would have larger in-degree scores (ie, the number of edges directed to the node). Our inductive analysis of keywords identified a number of key topic and subtopic areas: (1) COVID-19 pandemic; (2) mental health, including specific mental disorders; (3) e-mental health, including specific eHealth modalities and issues or challenges; (4) specific populations of interest; and (5) study field (eg, cancer and pediatrics). These are colorized separately in the figure to indicate their relation to one another.

### Mental Disorders of Focus

Although many of the 356 articles focused on mental health broadly, describing concerns relating to the effect of the pandemic on stress (n=78, 21.9%), loneliness or isolation (n=34, 9.6%), and general well-being (n=23, 6.5%), other studies focused their attention on specific mental disorders. A total of 71 of the 356 included articles (19.9%) were concerned with anxiety disorders (see Table 3 for examples), including the use of e-mental health to treat PTSD during the pandemic. A total of 48 articles were concerned with depression (13.5%). Other disorders of specific focus identified were addiction and substance abuse disorders (n=17, 4.8%), psychotic disorders (n=16, 4.5%), suicide (n=13, 3.7%), and eating disorders (n=11, 3.1%).

### eHealth Modalities

A large proportion of the 356 included articles described the application of e-mental health via telephone and/or videoconferencing during COVID-19 (n=142, 39.9%), as well as the use of smartphone apps (n=43, 12.1%) and support groups (n=6, 1.7%) in assisting people during times of need. For example, the review by Strudwick et al identified 31 smartphone apps and 114 web-based resources, including telephone support, virtual peer support groups, and discussion forums, that could be used to support the mental health of the Canadian public [58]. A smaller number of studies empirically tested the efficacy of specific e-mental health modalities during COVID-19. For example, a trial from Canada tested the efficacy of delivering videoconferencing psychotherapy for people with panic disorder and agoraphobia, demonstrating that cognitive behavioral therapy delivered via videoconferencing is no less effective than face-to-face delivery on all outcome measures, and provides important information to guide the delivery of e-mental health services during and after the COVID-19 crisis [47]. Less mentioned were virtual reality, wearables, and artificial intelligence, though these modalities were noted by Vadlamani et al as being the “future of telepsychiatry” [27].

### Specific Populations of Focus

A number of articles identified particular at-risk populations who would require greater care and/or more resources to overcome barriers to access e-mental health during the pandemic. A total of 90 articles out of 356 were focused on children and/or adolescents (25.3%) (see Table 3 for examples), and 39 articles focused on older adults or the elderly (5.6%) and veterans (4.8%) as at-risk populations.

There appears to be a general consensus in the literature that younger children and older adults, who may be less familiar with technology, may find it more difficult to access the benefits of e-mental health [59]. However, the flexible and remote nature of e-mental health may offer a more viable option to certain populations who may have historically fallen through the cracks or struggled to engage with traditional psychology service provision, for example, marginalized populations, such as those identifying as LGBTQ+ (lesbian, gay, bisexual, transgender, queer, questioning) [60], and young adults who prefer to seek help anonymously and at a time that suits them via smartphone apps [61].

A further 15 articles (4.2%) were specifically concerned with the mental health impact of COVID-19 on health professionals themselves. For example, Cheng et al outlined a peer support project offering support from mental health professionals overseas to frontline health care workers in Wuhan, China, via a popular smartphone app [62].

### Issues and Challenges

#### Overview

**Figure 7:**
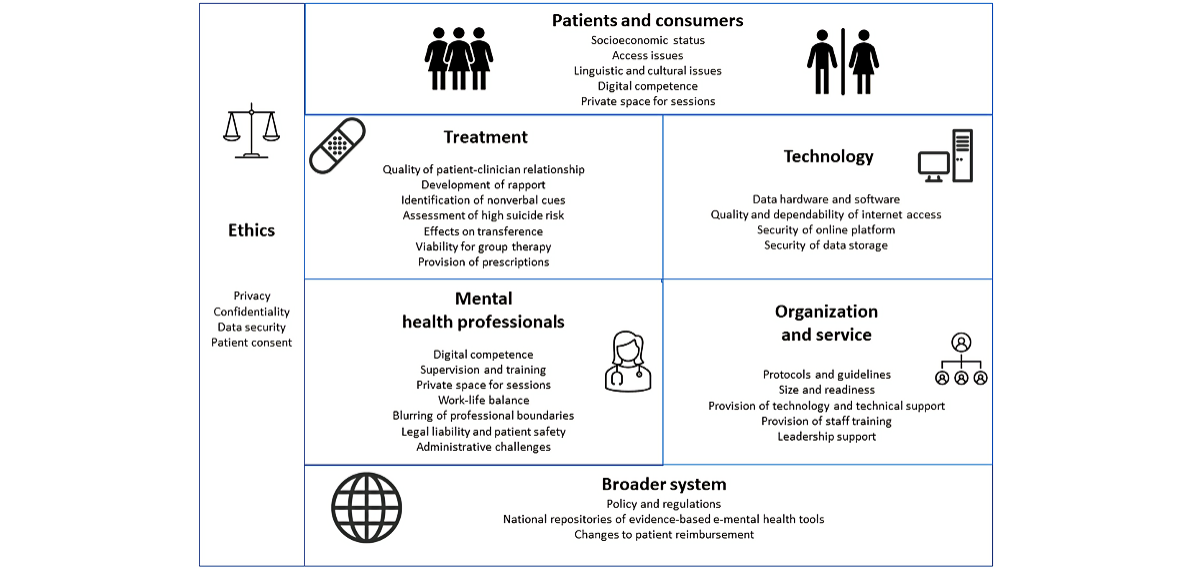
Summary of issues and challenges identified in the literature. e-mental health: electronic mental health.

A total of 68 papers out of 356 (19.1%) identified issues and challenges associated with e-mental health in the context of COVID-19. An inductive thematic analysis identified that many issues and challenges were highlighted, with complexities present at the patient, clinician, technological, treatment, ethical, organization or service, and broader system levels (see Figure 7 for a summary of the issues identified).

#### Privacy and Security Issues

At the technological level, included articles discussed how the rise in videoconferencing during COVID-19 has raised security concerns, as some platforms can be easily hacked or viewed by others [63,64]. Although some platforms, such as BlueJeans, have been developed with higher security measures in place (eg, offer end-to-end encryption), privacy cannot be guaranteed in its entirety. As a result of the COVID-19 pandemic, some governments issued emergency waivers suspending the requirement to comply with information protection laws in order to facilitate access to videoconferencing services [65]. However, patient confidentiality and privacy remains a compelling issue [27,64], encompassing informed consent procedures [66-68], risks associated with the blurring of professional boundaries [59], and concerns about family members listening in on sessions [64].

#### User Perspectives and Uptake

A mixed methods study on the perspectives and experiences of mental health professionals reported on their need for robust, secure, user-friendly technology and better logistical and technical support [69]. Issues have also been noted for clinicians who were insufficiently trained or had limited experience with technology [27]. An unstructured review article identified that rapid uptake of e-mental health technology has been facilitated by clinics providing clinician training as well as already having online storage, remote access to records, and videoconferencing technologies available in their workplace [70]. Larger clinics providing substantial services to the community reported the need to transition rapidly to ensure continuity of care, whereas smaller clinics were able to temporarily pause services or refer their clients elsewhere [70]. Articles also pointed to mental health professionals identifying concerns around the impact on the treatment relationship itself, noting the potential for inferior patient-clinician interactions [71,72] and therapeutic alliance [64,73,74]. They highlighted particular issues around the identification of nonverbal cues [59,66], transference processes [73,74], disturbances or interruptions to the session [27], and difficulties in assessing and responding appropriately to high-risk situations [59], particularly for children [75,76].

#### Access and Suitability

Despite most people now having internet access, the “digital divide” was still viewed as an issue in some articles for certain patient and consumer groups, such as the elderly, veterans, those of lower socioeconomic status, those with cognitive impairments or vision or hearing difficulties, and those still having access issues and poorer digital competence [27,63,64,72,77]. Concerns were also raised in some publications of patients with certain conditions not being suitable for e-mental health solutions, such as patients with psychosis, in acute crisis, or at risk of self-harm [63,64]. As identified by Naik et al, a “‘one-size-fits-all’ approach will not suit the needs of all patients,” with the need to refine e-mental health services to meet patient-specific needs (page 6) [74].

#### Policy and Regulation

At the broader system level, several articles pointed to changes in regulation and reimbursements that have enabled clinicians practicing in a wide range of settings to quickly adopt e-mental health solutions for both existing and new patients struggling with the pandemic’s impact [29-31,52,67]. Patel et al identified a number of local and national collaborative approaches to care that have been developed since COVID-19, such as “Webinars for Nursing Homes” that have linked palliative care, geriatrics, and psychiatric old-age services in Ireland through Project ECHO AIIHPC (Extension for Community Healthcare Outcomes, All Ireland Institute of Hospice and Palliative Care) to help nursing home staff improve their knowledge of e-mental health tools [51]. Patel et al further argued that a national repository of common e-mental health services could support collaboration between organizations.

## Discussion

### Principal Findings

Although it has been only 18 months since the start of the pandemic, this review identified that the total number of articles on e-mental health quickly thrived, with nonempirical articles peaking in June 2020 and empirical articles emerging later and becoming more common as the pandemic progressed. The location of articles was predominantly in high-income countries (ie, the United States, Australia, the United Kingdom, and Canada), reflecting general trends previously identified in the e-mental health literature [78,79]. However, a higher number of articles from medium-income countries were identified in this review in comparison to previous e-mental health reviews [78], with most of these articles coming from India and China. Increased outputs from China may be attributed to having the earliest outbreak of COVID-19, and for other middle-income countries, such as India, this could be reflective of the particular concerns and urgent solutions needed for people living in these countries [80].

Our bibliometric analysis indicated that the e-mental health literature during COVID-19 has had above-average influence, with almost half of the articles being ranked in the top 25% of all output scores by Altmetric and having an average CiteScore across articles of 4.22. Nonempirical articles (eg, commentaries or opinion pieces) received a notable amount of attention; articles of this type surged early in the pandemic, so elevated influence may reflect researchers and clinicians searching for new information on e-mental health, guidelines, evidence-based practice, and other relevant developments at an uncertain time and when empirical studies were limited.

The network analysis of author-supplied keywords identified key topic areas, which were explored further via our full-text analysis. Many articles focused broadly on mental health and well-being, with specific attention being paid to anxiety disorders, depression, substance abuse disorders, and psychosis. Children and adolescents were the most frequently identified in articles focused on specific populations, with other identified at-risk populations, including the elderly, veterans, and health professionals themselves. It was noted that younger children and older adults, who may be less familiar with technology, may find it more difficult to access the benefits of e-mental health. Frontline workers directly involved in the care of patients with COVID-19 have been identified as being at particularly high risk for mental health issues as a result of excessive workloads and work hours, insufficient protective equipment, feeling inadequately supported, as well as the high infection rate among medical staff [81]. Studies from past pandemics (eg, SARS) suggest that those in emergency departments, intensive care units, and infectious disease wards are at highest risk of developing adverse mental health outcomes [81].

Modalities of e-mental health largely focused on the application of “telehealth” via telephone or videoconferencing. However, there were also a number of articles reporting on the adoption of smartphone apps [82-85] and virtual support groups [62,86-88]. Self-directed treatment apps may be particularly helpful for those preferring to seek help anonymously and seeking flexibility or for those who have historically struggled to engage with traditional psychology service provision. Less mentioned were virtual reality, wearables, and artificial intelligence, though these modalities are widely believed as becoming more important in the future [27].

Since the start of the pandemic, both clinicians and patients have been required to gain both the skills and experience to adopt e-mental health solutions out of necessity, with a number of descriptive case studies being identified that inform the rapid conversion to e-mental health to continue to deliver care [89,90]. Various included articles articulated the main issues and challenges to this rapid conversion, as well as highlighting the fast-tracking of more relaxed regulations and supported reimbursements to overcome some of the barriers related to practicing virtual care. Included articles suggested that the experience has been largely valuable for patients and health care professionals alike; however, a number of concerns remain, with complexities identified at the levels of the patient, clinician, technology, treatment, ethics, organization or service, and broader system. A “one-size-fits-all” approach will not suit the needs of all patients, clinicians, or services [74]. Future research and interventions in e-mental health should investigate how to overcome or improve upon these barriers.

### Future Directions

After any subsequent waves of COVID-19 subside, the key question is whether we are likely to remain in the “new normal,” in which telehealth remains a prominent vehicle for mainstream mental health treatment delivery [66]. The ability of health care systems to continue to provide telehealth depends on the continuation of relaxed regulations and supported reimbursements to incentivize the use of telehealth [66]. It is unclear what rationale the regulatory bodies and insurers will employ to decide which prior limitations, if any, should be reinstated [66]. At the same time, given the large scale at which these tools are now being used, there is renewed urgency to assess and address ethical issues associated with e-mental health tools, such as maintaining privacy and patient data protection, and whether the privacy and safety regulations that have been relaxed need to be tightened to increase accountability [14].

Despite the swift adoption of the multitude of e-mental health apps and platforms that emerged during the pandemic, little is known about their immediate-, medium-, or long-term clinical value or the barriers to, and facilitators of, their uptake [18]. Given that many of the digital tools used in e-mental health have been developed outside of the health care system [91,92], there is also little understanding of how to optimize their integration into existing health care models and their various governance, funding, planning, and accountability frameworks. To date, there continues to be little translational research (ie, the implementation of e-mental health clinical trial research into practice settings) [93], limited use of quality evaluation measures [94], as well as little or no long-term follow-up [95]. This research-to-practice gap suggests there is much to learn about how e-mental health can be best incorporated into real-world settings to reap its benefits [96], with the current rapid uptake of e-mental health solutions providing a stimulus to addressing this gap.

Some key research questions identified in the literature include the need to compare specific outcomes for in-person versus remote care related to specific mental health conditions, such as those with paranoia or other psychotic disorders and substance use disorders. Such conditions may pose unique challenges to management via e-mental health programs and services [97,98], conditions that could also benefit from the greater flexibility of this modality [66,99]. Other questions have also emerged in the current literature: What types of patients respond particularly well to virtual rather than in-person visits? [14,72]; Are there differences in quality of care by socioeconomic status, health literacy, technology literacy, and other individual factors outside of psychiatric conditions?; What are the impacts of telehealth on the therapeutic relationship and on clinicians themselves?; and What are the financial implications of widespread adoption of telehealth? [14]. Now is the time to accelerate e-mental health research to ensure the continued success of virtual modes of care beyond the pandemic.

### Strengths and Limitations

The strengths of this review are the inclusion of a broad range of articles, including nonempirical articles, reviews, and protocols. In addition to looking at the countries of origin, we also examined article influence and provided a novel keyword analysis with inductive thematic coding to identify key topic areas. Our thorough analysis of the key issues and challenges identified gaps in research to guide the next wave of research on e-mental health. The limitations of this review are primarily methodological. We chose to use CiteScore data because almost 85% of the identified journals had CiteScore information and almost 70% had reported Altmetric data, but not all journals have CiteScore data, nor do all articles have Altmetric Attention Score values available. Similarly, the keyword analysis could only be conducted on journal articles that provided keywords. To supplement this, we undertook an analysis of specific subpopulations, mental disorders, and issues or challenges within titles and abstracts for all publications. Although we included a broad range of journal articles (eg, commentaries, reviews, letters to the editor, and protocols), we did not include a grey literature search, which may have broadened our understanding of the e-mental health field since the pandemic began. Although we identified a relatively high proportion of articles from medium-income countries, our restriction to records in English and published works may have underestimated the true amount of literature emerging from low- and middle-income countries. Although, the inclusion of non-English studies can add substantially to the resources required to complete a review, we believe it could be important to include them in an updated search of the literature in this field in the future. The inclusion of conference abstracts in the updated search would also be beneficial, given the rapidly emerging nature of the topic.

### Conclusions

Arguably, the COVID-19 pandemic is the defining moment for e-mental health adoption, with virtual care being likely to remain a prominent vehicle for mainstream mental health service delivery postpandemic. This review identified that many of the emerging e-mental health studies focused on the application of “telehealth” via telephone or videoconferencing, with notable interest in, and concern for, vulnerable populations, such as children, veterans, and health professionals as well as those with pre-existing mental health conditions, including anxiety disorders, depression, substance abuse disorders, and psychosis.

The rapid expansion of e-mental health services during the pandemic has been enabled by existing technology and swiftly implemented policies but has been challenged by numerous issues at the patient or clinician level and at the technology or treatment level, or due to factors at the ethical, organization or service, and broader system levels. Ensuring that the field advances beyond simply the sharp increase in publications documenting the use of e-mental health—and the concomitant interest by readers—to further accelerate access and quality of care beyond the pandemic will be the next big challenge.

## Appendix

Multimedia Appendix 1 Database search strategy using Ovid MEDLINE.

Multimedia Appendix 2 Coding structure and definitions for keyword classification.

Multimedia Appendix 3 Articles included in this study.

## Abbreviations

ECHO AIIHPC: Extension for Community Healthcare Outcomes, All Ireland Institute of Hospice and Palliative Care
e-mental health: electronic mental health
LGBTQ+: lesbian, gay, bisexual, transgender, queer, questioning
mHealth: mobile health
PRISMA-ScR: Preferred Reporting Items for Systematic Reviews and Meta-Analyses extension for Scoping Reviews
PTSD: posttraumatic stress disorder

## References

[1] (2017). Depression and Other Common Mental Disorders: Global Health Estimates.

[2] Fleischmann A, De Leo D (2014). The World Health Organization's report on suicide: A fundamental step in worldwide suicide prevention. Crisis.

[3] (2020). Report of the WHO-China Joint Mission on Coronavirus Disease 2019 (COVID-19).

[4] Guan W, Ni Z, Hu Y, Liang W, Ou C, He J, Liu L, Shan H, Lei C, Hui DSC, Du B, Li L, Zeng G, Yuen K, Chen R, Tang C, Wang T, Chen P, Xiang J, Li S, Wang J, Liang Z, Peng Y, Wei L, Liu Y, Hu Y, Peng P, Wang J, Liu J, Chen Z, Li G, Zheng Z, Qiu S, Luo J, Ye C, Zhu S, Zhong N, China Medical Treatment Expert Group for Covid-19 (2020). Clinical characteristics of coronavirus disease 2019 in China. N Engl J Med.

[5] Hale T, Anania J, Angrist N, Boby T, Cameron-Blake E, Di Folco M, Ellen L, Goldszmidt R, Hallas L, Kira B, Luciano M, Majumdar S, Nagesh R, Petherick A, Phillips T, Tatlow H, Webster S, Wood A, Zhang Y (2021). Variation in Government Responses to COVID-19. BSG-WP-2020/032. Version 12.0.

[6] Moreno C, Wykes T, Galderisi S, Nordentoft M, Crossley N, Jones N, Cannon M, Correll CU, Byrne L, Carr S, Chen EYH, Gorwood P, Johnson S, Kärkkäinen H, Krystal JH, Lee J, Lieberman J, López-Jaramillo C, Männikkö M, Phillips MR, Uchida H, Vieta E, Vita A, Arango C (2020). How mental health care should change as a consequence of the COVID-19 pandemic. Lancet Psychiatry.

[7] Gautam M, Thakrar A, Akinyemi E, Mahr G (2020). Current and future challenges in the delivery of mental healthcare during COVID-19. SN Compr Clin Med.

[8] OECD (2021). Tackling the Mental Health Impact of the COVID-19 Crisis: An Integrated, Whole-of-Society Response. OECD Policy Responses to Coronavirus (COVID-19).

[9] Izaguirre-Torres D, Siche R (2020). Covid-19 disease will cause a global catastrophe in terms of mental health: A hypothesis. Med Hypotheses.

[10] National Mental Health Commission (2019). Monitoring Mental Health and Suicide Prevention Reform. National Report 2019.

[11] Fisher JR, Tran TD, Hammarberg K, Sastry J, Nguyen H, Rowe H, Popplestone S, Stocker R, Stubber C, Kirkman M (2020). Mental health of people in Australia in the first month of COVID-19 restrictions: A national survey. Med J Aust.

[12] Bueno-Notivol J, Gracia-García P, Olaya B, Lasheras I, López-Antón R, Santabárbara J (2021). Prevalence of depression during the COVID-19 outbreak: A meta-analysis of community-based studies. Int J Clin Health Psychol.

[13] Ettman CK, Abdalla SM, Cohen GH, Sampson L, Vivier PM, Galea S (2020). Prevalence of depression symptoms in US adults before and during the COVID-19 pandemic. JAMA Netw Open.

[14] Martinez-Martin N, Dasgupta I, Carter A, Chandler JA, Kellmeyer P, Kreitmair K, Weiss A, Cabrera LY (2020). Ethics of digital mental health during COVID-19: Crisis and opportunities. JMIR Ment Health.

[15] Barak A, Grohol JM (2011). Current and future trends in internet-supported mental health interventions. J Technol Hum Serv.

[16] Christensen H, Hickie IB (2010). E-mental health: A new era in delivery of mental health services. Med J Aust.

[17] Stone L, Waldron R (2019). "Great Expectations" and e-mental health: The role of literacy in mediating access to mental healthcare. Aust J Gen Pract.

[18] Research Futures (2021). e-Mental health implementation requires more robust studies. Research Futures.

[19] Fatehi F, Wootton R (2012). Telemedicine, telehealth or e-health? A bibliometric analysis of the trends in the use of these terms. J Telemed Telecare.

[20] Martin-Khan M, Freeman S, Adam K, Betkus G, Marston HR, Freeman S, Musselwhite C (2017). The evolution of telehealth. Mobile e-Health.

[21] Abbott PA, Liu Y (2013). A scoping review of telehealth. Yearb Med Inform.

[22] Rush KL, Howlett L, Munro A, Burton L (2018). Videoconference compared to telephone in healthcare delivery: A systematic review. Int J Med Inform.

[23] Gratzer D, Torous J, Lam RW, Patten SB, Kutcher S, Chan S, Vigo D, Pajer K, Yatham LN (2021). Our digital moment: Innovations and opportunities in digital mental health care. Can J Psychiatry.

[24] Torous J, Keshavan M (2020). COVID-19, mobile health and serious mental illness. Schizophr Res.

[25] (2020). Medicare telemedicine health care provider fact sheet. Centers for Medicare & Medicaid Services.

[26] Chin HP, Palchik G (2021). Telepsychiatry in the age of COVID: Some ethical considerations. Camb Q Healthc Ethics.

[27] Vadlamani LN, Sharma V, Emani A, Gowda MR (2020). Telepsychiatry and outpatient department services. Indian J Psychol Med.

[28] Dinakaran D, Basavarajappa C, Manjunatha N, Kumar CN, Math SB (2020). Telemedicine practice guidelines and telepsychiatry operational guidelines, India-A commentary. Indian J Psychol Med.

[29] Jayarajan D, Sivakumar T, Torous JB, Thirthalli J (2020). Telerehabilitation in psychiatry. Indian J Psychol Med.

[30] (2021). Wellness Together Canada.

[31] Tricco AC, Lillie E, Zarin W, O'Brien KK, Colquhoun H, Levac D, Moher D, Peters MDJ, Horsley T, Weeks L, Hempel S, Akl EA, Chang C, McGowan J, Stewart L, Hartling L, Aldcroft A, Wilson MG, Garritty C, Lewin S, Godfrey CM, Macdonald MT, Langlois EV, Soares-Weiser K, Moriarty J, Clifford T, Tunçalp Ö, Straus SE (2018). PRISMA Extension for Scoping Reviews (PRISMA-ScR): Checklist and explanation. Ann Intern Med.

[32] Peters MDJ, Marnie C, Tricco AC, Pollock D, Munn Z, Alexander L, McInerney P, Godfrey CM, Khalil H (2020). Updated methodological guidance for the conduct of scoping reviews. JBI Evid Synth.

[33] (2015). The Joanna Briggs Institute Reviewers’ Manual 2015: Methodology for JBI Scoping Reviews.

[34] Paré G, Trudel M, Jaana M, Kitsiou S (2015). Synthesizing information systems knowledge: A typology of literature reviews. Inf Manag.

[35] Pham MT, Rajić A, Greig JD, Sargeant JM, Papadopoulos A, McEwen SA (2014). A scoping review of scoping reviews: Advancing the approach and enhancing the consistency. Res Synth Methods.

[36] Altman DG (1991). Practical Statistics for Medical Research.

[37] Fang Z, Costas R (2020). Studying the accumulation velocity of altmetric data tracked by Altmetric.com. Scientometrics.

[38] Roldan-Valadez E, Salazar-Ruiz SY, Ibarra-Contreras R, Rios C (2019). Current concepts on bibliometrics: A brief review about impact factor, Eigenfactor score, CiteScore, SCImago Journal Rank, Source-Normalised Impact per Paper, H-index, and alternative metrics. Ir J Med Sci.

[39] Thelwall M, Nevill T (2018). Could scientists use Altmetric.com scores to predict longer term citation counts?. J Informetr.

[40] (2021). World Bank country and lending groups. The World Bank.

[41] Bastian M, Heymann S, Jacomy M (2009). Gephi: An open source software for exploringmanipulating networks. Proceedings of the 3rd International AAAI Conference on Weblogs Social Media.

[42] Ben-Zeev D, Buck B, Meller S, Hudenko WJ, Hallgren KA (2020). Augmenting evidence-based care with a texting mobile interventionist: A pilot randomized controlled trial. Psychiatr Serv.

[43] Alessi J, de Oliveira GB, Franco DW, Becker AS, Knijnik CP, Kobe GL, Amaral BB, de Brito A, Schaan BD, Telo GH (2021). Telehealth strategy to mitigate the negative psychological impact of the COVID-19 pandemic on type 2 diabetes: A randomized controlled trial. Acta Diabetol.

[44] Watts S, Marchand A, Bouchard S, Gosselin P, Langlois F, Belleville G, Dugas MJ (2020). Telepsychotherapy for generalized anxiety disorder: Impact on the working alliance. J Psychother Integr.

[45] Kubo A, Kurtovich E, McGinnis M, Aghaee S, Altschuler A, Quesenberry C, Kolevska T, Liu R, Greyz-Yusupov N, Avins A (2020). Pilot pragmatic randomized trial of mHealth mindfulness-based intervention for advanced cancer patients and their informal caregivers. Psychooncology.

[46] Rodriguez-Villa E, Naslund J, Keshavan M, Patel V, Torous J (2020). Making mental health more accessible in light of COVID-19: Scalable digital health with digital navigators in low and middle-income countries. Asian J Psychiatr.

[47] Bouchard S, Allard M, Robillard G, Dumoulin S, Guitard T, Loranger C, Green-Demers I, Marchand A, Renaud P, Cournoyer L, Corno G (2020). Videoconferencing psychotherapy for panic disorder and agoraphobia: Outcome and treatment processes from a non-randomized non-inferiority trial. Front Psychol.

[48] Puspitasari AJ, Heredia D, Coombes BJ, Geske JR, Gentry MT, Moore WR, Sawchuk CN, Schak KM (2021). Feasibility and initial outcomes of a group-based teletherapy psychiatric day program for adults with serious mental illness: Open, nonrandomized trial in the context of COVID-19. JMIR Ment Health.

[49] Pierce BS, Perrin PB, Tyler CM, McKee GB, Watson JD (2021). The COVID-19 telepsychology revolution: A national study of pandemic-based changes in US mental health care delivery. Am Psychol.

[50] Steinberg DM, Schneider NM, Guler J, Garcia AM, Kullgren KA, Agoston AM, Mudd E, Carter BD, Judd-Glossy L (2021). Pediatric consultation-liaison psychology services during the COVID-19 pandemic: Pivoting to provide care. Clin Pract Pediatr Psychol.

[51] Patel S, Gannon A, Dolan C, McCarthy G (2020). Telehealth in psychiatry of old age: Ordinary care in extraordinary times in rural north-west Ireland. Am J Geriatr Psychiatry.

[52] Haque SN (2021). Telehealth beyond COVID-19. Psychiatr Serv.

[53] Patra S, Patro BK (2020). COVID-19 and adolescent mental health in India. Lancet Psychiatry.

[54] Soron TR, Shariful Islam SM, Ahmed HU, Ahmed SI (2020). The hope and hype of telepsychiatry during the COVID-19 pandemic. Lancet Psychiatry.

[55] Rahman A, Naslund JA, Betancourt TS, Black CJ, Bhan A, Byansi W, Chen H, Gaynes BN, Restrepo CG, Gouveia L, Hamdani SU, Marsch LA, Petersen I, Bahar OS, Shields-Zeeman L, Ssewamala F, Wainberg ML (2020). The NIMH global mental health research community and COVID-19. Lancet Psychiatry.

[56] Kozloff N, Mulsant BH, Stergiopoulos V, Voineskos AN (2020). The COVID-19 global pandemic: Implications for people with schizophrenia and related disorders. Schizophr Bull.

[57] Zhou X, Snoswell CL, Harding LE, Bambling M, Edirippulige S, Bai X, Smith AC (2020). The role of telehealth in reducing the mental health burden from COVID-19. Telemed J E Health.

[58] Strudwick G, Sockalingam S, Kassam I, Sequeira L, Bonato S, Youssef A, Mehta R, Green N, Agic B, Soklaridis S, Impey D, Wiljer D, Crawford A (2021). Digital interventions to support population mental health in Canada during the COVID-19 pandemic: Rapid review. JMIR Ment Health.

[59] Payne L, Flannery H, Kambakara Gedara C, Daniilidi X, Hitchcock M, Lambert D, Taylor C, Christie D (2020). Business as usual? Psychological support at a distance. Clin Child Psychol Psychiatry.

[60] Craig SL, Iacono G, Pascoe R, Austin A (2021). Adapting clinical skills to telehealth: Applications of affirmative cognitive-behavioral therapy with LGBTQ+ youth. Clin Soc Work J.

[61] Han J, McGillivray L, Wong QJ, Werner-Seidler A, Wong I, Calear A, Christensen H, Torok M (2020). A mobile health intervention (LifeBuoy app) to help young people manage suicidal thoughts: Protocol for a mixed-methods randomized controlled trial. JMIR Res Protoc.

[62] Cheng P, Xia G, Pang P, Wu B, Jiang W, Li Y, Wang M, Ling Q, Chang X, Wang J, Dai X, Lin X, Bi X (2020). COVID-19 epidemic peer support and crisis intervention via social media. Community Ment Health J.

[63] Looi JC, Pring W (2020). Private metropolitan telepsychiatry in Australia during Covid-19: Current practice and future developments. Australas Psychiatry.

[64] Di Carlo F, Sociali A, Picutti E, Pettorruso M, Vellante F, Verrastro V, Martinotti G, di Giannantonio M (2021). Telepsychiatry and other cutting-edge technologies in COVID-19 pandemic: Bridging the distance in mental health assistance. Int J Clin Pract.

[65] Wright JH, Caudill R (2020). Remote treatment delivery in response to the COVID-19 pandemic. Psychother Psychosom.

[66] Chen JA, Chung W, Young SK, Tuttle MC, Collins MB, Darghouth SL, Longley R, Levy R, Razafsha M, Kerner JC, Wozniak J, Huffman JC (2020). COVID-19 and telepsychiatry: Early outpatient experiences and implications for the future. Gen Hosp Psychiatry.

[67] Arias F, Safi DE, Miranda M, Carrión CI, Diaz Santos AL, Armendariz V, Jose IE, Vuong KD, Suarez P, Strutt AM, STAR Consortium (2020). Teleneuropsychology for monolingual and bilingual Spanish-speaking adults in the time of COVID-19: Rationale, professional considerations, and resources. Arch Clin Neuropsychol.

[68] Stoll J, Sadler JZ, Trachsel M (2020). The ethical use of telepsychiatry in the Covid-19 pandemic. Front Psychiatry.

[69] Feijt M, de Kort Y, Bongers I, Bierbooms J, Westerink J, IJsselsteijn W (2020). Mental health care goes online: Practitioners' experiences of providing mental health care during the COVID-19 pandemic. Cyberpsychol Behav Soc Netw.

[70] Hames JL, Bell DJ, Perez-Lima LM, Holm-Denoma JM, Rooney T, Charles NE, Thompson SM, Mehlenbeck RS, Tawfik SH, Fondacaro KM, Simmons KT, Hoersting RC (2020). Navigating uncharted waters: Considerations for training clinics in the rapid transition to telepsychology and telesupervision during COVID-19. J Psychother Integr.

[71] Uscher-Pines L, Sousa J, Raja P, Mehrotra A, Barnett ML, Huskamp HA (2020). Suddenly becoming a "virtual doctor": Experiences of psychiatrists transitioning to telemedicine during the COVID-19 pandemic. Psychiatr Serv.

[72] Held P, Klassen BJ, Coleman JA, Thompson K, Rydberg TS, Van Horn R (2021). Delivering intensive PTSD treatment virtually: The development of a 2-week intensive cognitive processing therapy-based program in response to COVID-19. Cogn Behav Pract.

[73] Cowan A, Johnson R, Close H (2020). Telepsychiatry in psychotherapy practice. Innov Clin Neurosci.

[74] Naik SS, Manjunatha N, Kumar CN, Math SB, Moirangthem S (2020). Patient's perspectives of telepsychiatry: The past, present and future. Indian J Psychol Med.

[75] Racine N, Hartwick C, Collin-Vézina D, Madigan S (2020). Telemental health for child trauma treatment during and post-COVID-19: Limitations and considerations. Child Abuse Negl.

[76] Holland M, Hawks J, Morelli LC, Khan Z (2021). Risk assessment and crisis intervention for youth in a time of telehealth. Contemp Sch Psychol.

[77] Sivakumar PT, Mukku SSR, Kar N, Manjunatha N, Phutane VH, Sinha P, Kumar CN, Math SB (2020). Geriatric telepsychiatry: Promoting access to geriatric mental health care beyond the physical barriers. Indian J Psychol Med.

[78] Yang Y, Iqbal U, Ching JH, Ting JB, Chiu H, Tamashiro H, Hsu YE (2015). Trends in the growth of literature of telemedicine: A bibliometric analysis. Comput Methods Programs Biomed.

[79] Whan P, Brown NA, Wootton R (2016). A bibliographic snapshot of the telemedicine citation literature. J Telemed Telecare.

[80] Kola L, Kohrt BA, Hanlon C, Naslund JA, Sikander S, Balaji M, Benjet C, Cheung EYL, Eaton J, Gonsalves P, Hailemariam M, Luitel NP, Machado DB, Misganaw E, Omigbodun O, Roberts T, Salisbury TT, Shidhaye R, Sunkel C, Ugo V, van Rensburg AJ, Gureje O, Pathare S, Saxena S, Thornicroft G, Patel V (2021). COVID-19 mental health impact and responses in low-income and middle-income countries: Reimagining global mental health. Lancet Psychiatry.

[81] Spoorthy MS, Pratapa SK, Mahant S (2020). Mental health problems faced by healthcare workers due to the COVID-19 pandemic-A review. Asian J Psychiatr.

[82] Alexopoulos AR, Hudson JG, Otenigbagbe O (2020). The use of digital applications and COVID-19. Community Ment Health J.

[83] Psihogios AM, Stiles-Shields C, Neary M (2020). The needle in the haystack: Identifying credible mobile health apps for pediatric populations during a pandemic and beyond. J Pediatr Psychol.

[84] Lewis M, Palmer VJ, Kotevski A, Densley K, O'Donnell ML, Johnson C, Wohlgezogen F, Gray K, Robins-Browne K, Burchill L (2021). Rapid design and delivery of an experience-based co-designed mobile app to support the mental health needs of health care workers affected by the COVID-19 pandemic: Impact evaluation protocol. JMIR Res Protoc.

[85] McDonnell A, MacNeill C, Chapman B, Gilbertson N, Reinhardt M, Carreiro S (2021). Leveraging digital tools to support recovery from substance use disorder during the COVID-19 pandemic response. J Subst Abuse Treat.

[86] Datlen GW, Pandolfi C (2020). Developing an online art therapy group for learning disabled young adults using WhatsApp. Int J Art Ther.

[87] Fortuna KL, Myers AL, Walsh D, Walker R, Mois G, Brooks JM (2020). Strategies to increase peer support specialists' capacity to use digital technology in the era of COVID-19: Pre-post study. JMIR Ment Health.

[88] Viswanathan R, Myers MF, Fanous AH (2020). Support groups and individual mental health care via video conferencing for frontline clinicians during the COVID-19 pandemic. Psychosomatics.

[89] Yellowlees P, Nakagawa K, Pakyurek M, Hanson A, Elder J, Kales HC (2020). Rapid conversion of an outpatient psychiatric clinic to a 100% virtual telepsychiatry clinic in response to COVID-19. Psychiatr Serv.

[90] Sullivan AB, Kane A, Roth AJ, Davis BE, Drerup ML, Heinberg LJ (2020). The COVID-19 crisis: A mental health perspective and response using telemedicine. J Patient Exp.

[91] Batterham PJ, Sunderland M, Calear AL, Davey CG, Christensen H, Teesson M, Kay-Lambkin F, Andrews G, Mitchell PB, Herrman H, Butow PN, Krouskos D (2015). Developing a roadmap for the translation of e-mental health services for depression. Aust N Z J Psychiatry.

[92] Ellis LA, Augustsson H, Grødahl AI, Pomare C, Churruca K, Long JC, Ludlow K, Zurynski YA, Braithwaite J (2020). Implementation of e-mental health for depression and anxiety: A critical scoping review. J Community Psychol.

[93] Wozney L, McGrath PJ, Gehring ND, Bennett K, Huguet A, Hartling L, Dyson MP, Soleimani A, Newton AS (2018). eMental healthcare technologies for anxiety and depression in childhood and adolescence: Systematic review of studies reporting implementation outcomes. JMIR Ment Health.

[94] Kidholm K, Ekeland AG, Jensen LK, Rasmussen J, Pedersen CD, Bowes A, Flottorp SA, Bech M (2012). A model for assessment of telemedicine applications: MAST. Int J Technol Assess Health Care.

[95] Christensen H, Proudfoot J, Woodward A, Hosie A, Klein B, Morgan C (2014). E-mental Health Services in Australia 2014: Current and Future. Working Paper.

[96] Ossebaard HC, Van Gemert-Pijnen L (2016). eHealth and quality in health care: Implementation time. Int J Qual Health Care.

[97] Mohan A, Ambekar A (2020). Telepsychiatry and addiction treatment. Indian J Psychol Med.

[98] DeLuca JS, Andorko ND, Chibani D, Jay SY, Rakhshan Rouhakhtar PJ, Petti E, Klaunig MJ, Thompson EC, Millman ZB, Connors KM, Akouri-Shan L, Fitzgerald J, Redman SL, Roemer C, Bridgwater MA, DeVylder JE, King CA, Pitts SC, Reinblatt SP, Wehring HJ, Bussell KL, Solomon N, Edwards SM, Reeves GM, Buchanan RW, Schiffman J (2020). Telepsychotherapy with youth at clinical high risk for psychosis: Clinical issues and best practices during the COVID-19 pandemic. J Psychother Integr.

[99] Miu AS, Vo HT, Palka JM, Glowacki CR, Robinson RJ (2020). Teletherapy with serious mental illness populations during COVID-19: Telehealth conversion and engagement. Couns Psychol Q.

