# Supplementary material for: The Application of e-Mental Health in Response to COVID-19: Scoping Review and Bibliometric Analysis
Source: JMIR Ment Health. 2021 Dec 6;8(12):e32948. doi: 10.2196/32948 (PMC8651237; doi:10.2196/32948)
Supplement: Multimedia Appendix 1 [file mental_v8i12e32948_app1.docx]

**Multimedia Appendix 1**

**Database search strategy: Ovid MEDLINE**

| Number | Search terms |
| --- | --- |
| 1 | Telemedicine/ |
| 2 | (eHealth or telehealth or mobile health or mHealth or telemedicine or telehealth).mp. |
| 3 | 1 or 2 |
| 4 | Mental Health/ or Anxiety/ or Depression/ or Depressive disorder/ or Mood disorders/ |
| 5 | (Mental Health or psychiatr* or Anxi* or depress* or depressive disorder or stress disorder* or mood disorder*).mp. |
| 6 | 4 or 5 |
| 7 | (e-mental health or emental health or e-psychiatr* or m-mental health or mmental health or  tele-psychiatr* or telepsychiatr* or telemental health* or tele-mental health*).mp. |
| 8 | COVID-19/ or SARS-CoV-2/ |
| 9 | (COVID-19 or SARS-CoV-2 or severe acute respiratory syndrome coronavirus 2 or 2019nCOV or HCoV-19).mp. |
| 10 | 8 or 9 |
| 11 | 3 and 6 and 10 |
| 12 | 7 and 10 |
| 13 | 11 or 12 |

Note. * symbolises truncation
