# Supplementary material for: The Application of e-Mental Health in Response to COVID-19: Scoping Review and Bibliometric Analysis
Source: JMIR Ment Health. 2021 Dec 6;8(12):e32948. doi: 10.2196/32948 (PMC8651237; doi:10.2196/32948)
Supplement: Multimedia Appendix 2 [file mental_v8i12e32948_app2.docx]

**Multimedia Appendix 2**

**Coding structure and definitions for keyword classification**

| **Theme** | **Subtheme** |
| --- | --- |
| Pandemic | Includes all keywords related to the Covid-19 pandemic   - Covid-19 - Pandemic - SARS-CoV-2 - SARS virus |
| Mental health | Includes all keywords related to mental health and wellbeing and specific mental health disorders.  Subtheme: Mental health and well-being   - Mental health - Stress or Distress - Well-being   Subtheme: Mental health disorders   - Anxiety - Depression - Trauma or Post-traumatic stress disorder (PTSD) - Addiction or Substance abuse disorder - Autism spectrum disorder - Eating disorders - Schizophrenia |
| e-mental health | Includes all keywords related to the use of information and communication for improving health. Keywords specifically related to mental health are placed under e-mental health subtheme  Subtheme: Mental health and well-being   - Telehealth - Telemedicine - Telepsychiatry - mhealth - e-health - Telepsychotherapy - Digital health   Subtheme: e-mental health   - Tele-mental health - Digital mental health - e-mental health   Subtheme: e-mental modalities   - Mobile apps - Apps - Videoconferencing - Internet - Social media - Smartphone apps   Subtheme: Issues/challenges   - Ethics - Privacy - Disparities |
| Population | Includes all keywords related to specific populations of interest.   - Veterans - Adolescents or Young adult - Aged - Healthcare workers - Children - Youth |
| Field | Includes all keywords related to specific disciplines, medicine specialities or therapies.  Subtheme: Discipline   - Psychiatry - Public health - Psychology   Subtheme: Medicine specialty   - Cancer - Oncology - Psycho-oncology   Subtheme: Therapy   - Psychotherapy - Access to care - CBT - Therapeutic alliance |
